# Correction: Shoaib et al. Neuroprotective Effects of Dried Tubers of *Aconitum napellus*. *Plants* 2020, *9*, 356

**DOI:** 10.3390/plants11010080

**Published:** 2021-12-28

**Authors:** Ambreen Shoaib, Hefazat Hussain Siddiqui, Rakesh Kumar Dixit, Sahabjada Siddiqui, Badrud Deen, Andleeb Khan, Salman H. Alrokayan, Haseeb A. Khan, Parvaiz Ahmad

**Affiliations:** 1 Department of Clinical Pharmacy, Faculty of Pharmacy, Jazan University, Jazan 45142, Saudi Arabia; amber8739@yahoo.com; 2 Department of Pharmacology, Faculty of Pharmacy, Integral University, Lucknow 226026, India; hefazats@hotmail.com; 3 Department of Pharmacology, King George Medical University, Lucknow 226003, India; dixitkumarrakesh@gmail.com; 4 Department of Biotechnology, Era’s Lucknow Medical College & Hospital, Era University, Lucknow 226003, India; sahabjadabiotech04@gmail.com; 5 Department of Pharmacology & Toxicology, Faculty of Pharmacy, Jazan University, Jazan 45142, Saudi Arabia; andleeb.tox@gmail.com; 6 Department of Biochemistry, College of Science, King Saud University, Riyadh 11451, Saudi Arabia; salrokayan@ksu.edu.sa (S.H.A.); khan_haseeb@yahoo.com (H.A.K.); 7 Botany and Microbiology Department, College of Science, King Saudi University, Riyadh 11451, Saudi Arabia

We are sorry to report that some images in Figure 1 reported in our recently published paper [1] were incorrect. There was an error during the formatting of text and the grouping of different frames of images into one single block. We wish to change Figure 1 from:

**Figure 1:**
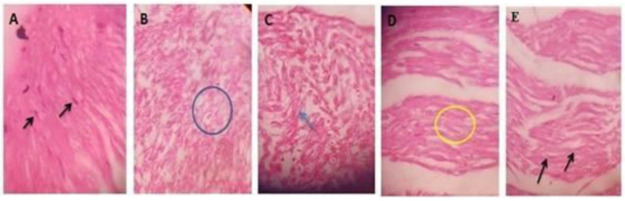
A hematoxylin and eosin section of sciatic nerve shows (**A**) Control animal shows treated with 0.5% CMC with normal arrangement of nerve fibers with elongated Schwann cells. (**B**) Diabetic control group shows axonal swelling. (**C**) Treated with GMT-2.5 shows axonal swelling of nerve fibers. (**D**) This group treated with GMT-5 showing the mild derangements of nerve fibers. (**E**) pregabalin treated group shows uniformly arranged nerve fibers.

to:

**Figure 1:**
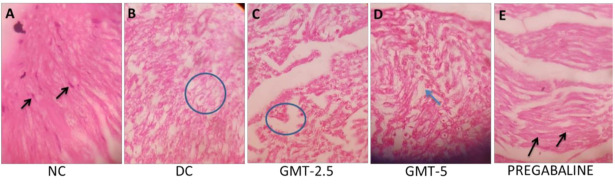
A hematoxylin and eosin section of sciatic nerve tissue (**A**) Control group treated with 0.5% CMC showing the normal arrangement of nerve fibers with elongated Schwann cells. (**B**) Diabetic control group showing axonal swelling. (**C**) Treated with GMT-2.5 showing axonal swelling of nerve fibers. (**D**) Treated with GMT-5 showing the mild derangements of nerve fibers. (**E**) Treated with pregabalin group showing uniformly arranged nerve fibers.

This change has no impact on the caption of Figure 1 and other content of the paper. We would like to apologize for any inconvenience caused to the readers. This Correction was approved with consultation with an Editorial Board member and the Editor-in-Chief.

## References

[1] Shoaib A., Siddiqui H.H., Dixit R.K., Siddiqui S., Deen B., Khan A., Alrokayan S.H., Khan H.A., Ahmad P. (2020). Neuroprotective Effects of Dried Tubers of *Aconitum napellus*. Plants.

